# Storage-Efficient 16-Bit Hybrid IP Traceback with Single Packet

**DOI:** 10.1155/2014/659894

**Published:** 2014-10-20

**Authors:** Ming Hour Yang

**Affiliations:** Information and Computer Engineering, Chung Yuan Christian University, 200 Chung Pei Road, Chung Li City, Taoyuan County 32023, Taiwan

## Abstract

Since adversaries may spoof their source IPs in the attacks, traceback schemes have been proposed to identify the attack source. However, some of these schemes' storage requirements increase with packet numbers. Some even have false positives because they use an IP header's fragment offset for marking. Thus, we propose a 16-bit single packet hybrid IP traceback scheme that combines packet marking and packet logging with high accuracy and low storage requirement. The size of our log tables can be bounded by route numbers. We also set a threshold to determine whether an upstream interface number is stored in a log table or in a marking field, so as to balance the logging frequency and our computational loads. Because we store user interface information on small-degree routers, compared with current single packet traceback schemes, ours can have the lowest storage requirements. Besides, our traceback achieves zero false positive/negative rates and guarantees reassembly of fragmented packets at the destination.

## 1. Introduction

Recent years have seen the rapid growth of the Internet, and the widespread Internet services have become a part of our daily life. These services, however, are vulnerable to many potential threats. To name one out of many, a malicious user may launch distributed/denial of service (D/DoS) attacks to disrupt the Internet services. Judging from the number of attacking packets, a D/DoS attack can be categorized into flooding-based attack and software exploit attack [1]. In a flooding-based attack, the victim's resources can be exhausted by a huge amount of forged source packets. But in a software exploit attack, a villain needs to find the host's vulnerabilities and then uses only a few packets to launch attacks, for example, Teardrop attacks and LAND attacks [2]. Normally the source and destination IP addresses are stored in a packet's IP header to indicate its source and destination. In practice, however, most routers do not verify a packet's source IP. This is why attackers usually take this advantage and spoof their real address to evade tracking. This security issue has come to our attention and we find it urgent to propose an efficient traceback scheme tracking the real source of impersonation attacks.

Therefore, packet-marking schemes are proposed to trace the real source of flooding-based packets. They use the free fields of each packet's IP header to mark the packet's route and the routers along the route. As these packets are usually in a huge amount, these marking schemes are categorized as probabilistic packet marking (PPM) [3–9] and deterministic packet marking (DPM) [10–14]. The two methods are proposed to lower the routers' marking loads. While PPM is purely based on probability, DPM puts a single mark on inbound packets at the point of network ingress. However, both PPM and DPM require at least eight packets for path reconstruction [12], so they may not be able to trace the source of software exploit attacks, which can use only one packet to paralyze the system.

If we want to achieve single packet traceback, we have to use packet logging schemes [15–17] to log the packet's unchanged data on the routers. And the path reconstruction requires hop-by-hop queries of previous routers. For example, in Snoeren et al.'s SPIE [15] and Zhang and Guan's TOPO [16], routers that comply with these schemes have to use a Bloom filter [18] to log their packets' digests. But if the filter logs too many packets, there might be collision in their log tables and therefore they will have false positives during path reconstruction. In order to reduce the chance of collision and keep the false positive rate within 1%, SPIE has to back up and refresh its log tables when the accumulative packet size is larger than the table's size by 20% [15]. Likewise, TOPO [16] uses each upstream router's identifier to decrease the chance of collision and false positives. However, like SPIE, TOPO still has to clear its logged data on the routers when the packet number is too large. If the log tables are refreshed, the traceback scheme is unable to reconstruct the attack route.

For these reasons, hybrid single packet traceback schemes have been proposed to combine packet marking and packet logging. These methods can achieve single packet tracking and have lower storage requirements and false positive rates. There are two types of these hybrid single packet traceback schemes: the first type hashes each packet's marking field (or some specific fields of a packet's header) to compute an index and modify the indexed value in the Bloom filter [19–23]. But the storage requirement on each router grows when the packet number increases. When it exceeds the router's quota, the logged data will be refreshed and the path reconstruction fails.

The other type encodes a packet's route as a mark and stores it in the packet's header. If the mark is larger than the size of a marking field, the packet's route is logged onto a router [24–26] to decrease each router's storage loads. These schemes decrease the false negative rate because the logged data in a router does not need to be refreshed. Lu et al. use multiprotocol label switching (MPLS) networks [25] to encode a router's upstream routes and the destination router's ID as a 29-bit mark. When a router receives the packet, it uses the packet's destination IP as an index to choose a log table to log this mark. Then the router writes its ID and the packet's upstream routes into the mark, so that the downstream routers can use the mark to trace the origin of the attack. However, in Lu et al.'s scheme, every router that the packet passes through has to log the packet's mark. Besides, the scheme does not have indexes for their log tables. It needs to do an exhaustive search during path reconstruction, so as to find the corresponding upstream interface number of the attack packet. Since the exhaustive search consumes lots of computation power of a router, it makes their traceback scheme not practical. Hence, M. H. Yang and M. C. Yang propose RIHT [24] to encode all the upstream routers' interface numbers as their log table's indexes. The routers do not need to search their log tables during path reconstruction. In their scheme, the maximum storage requirement for each router is only 320 KB, and their average storage requirement can remain low because they do one logging on every three routers.

Besides, RIHT's marking field requires 32 bits and consists of an IP header's ID, Fragment Flags, and Fragment Offset [24], whereas Lu et al. use ID and Fragment Offset as their 29-bit marking field. But in the two schemes, if a packet's size exceeds the maximum transmission unit (MTU), the packet will be fragmented and cannot be assembled at the destination. Thus Yang proposes a 16-bit traceback scheme (Hybrid Single-Packet IP Traceback with Low Storage and High Accuracy, HAHIT) [26] using only the ID field as their marking field. If a router receives a packet whose mark is larger than 65535, the router hashes the packet's destination IP and uses the hash value to assign a log table; it also hashes the packet's mark to compute an index value. According to the table number and the index value, the packet's route is logged on the router. Unlike RIHT [24] and Lu et al.'s scheme [25], Yang's scheme [26] prevents the fragmented packets from being dropped and guarantees precision in the traceback of an attack. Besides, because a router that supports IPsec may need to add ESP's header to each packet, it can increase a packet's length and the chance of fragmentation. According to John and Tafvelln's research [27], 63% fragmented packets are ESP packets. Hence, IPsec may not work because of the high chance of packet fragmentation and because of the difficulty in packet reassembly. Also, the values of Fragment Flag and Fragment Offset are used to show whether a packet is fragmented or not. If they are used as a marking field instead, the downstream router cannot tell if the received packet has been fragmented. Further, RIHT and Lu et al.'s method may have a packet-drop issue because, according to RFC 6274 [28], when the value of Fragment Offset is larger than a packet's maximum length, the packet will be dropped.

However, in Yang's 16-bit hybrid single IP traceback scheme [26], he uses the quadratic probing algorithm to search an available index for his log tables and to minimize the impact of collision. In quadratic probing, the load factor suggests the usage rate of each log table. RIHT defines its load factor according to the chance of their successful and unsuccessful searches, and it finds its unsuccessful search rate soars when each log table has used over half its slots. In order to balance the collision times and each table's usage rate, Yang sets his load factor as 0.5. However, the use of quadratic probing has caused half of his log tables to be unused and this results in a waste of space to the routers. To reduce the storage requirements for logging, we propose two schemes in our 16-bit hybrid traceback protocol to encode the upstream routers' interface numbers as an index of the log table's entry. A router will compare its degrees with a threshold to choose a coding scheme to calculate the mark. Therefore, we can decrease the storage requirements by reducing the logging frequency. Also, we propose a logging scheme to further reduce the storage requirements for logging. To write the packet's route into a log table, we search the first empty slot in the log table from the top to the bottom sequentially. The main contributions of our scheme are listed below and we aim to satisfy the first three so as to achieve the last two:

- Single IP traceback.
- The storage requirements of logging are bounded by the number of upstream routes, and no duplicate route is logged.
- The fragmented packets can be reassembled at the destination.
- Reduction of the logging frequency.
- Decrease of the storage requirements of logging.

Our traceback scheme will be elaborated in the following sections. The simulation, analysis, and comparison of our scheme and other related hybrid traceback approaches are provided in Section 3. And a conclusion is drawn in Section 4.

## 2. Single Packet IP Traceback Protocol

In order to prevent packet drop caused by fragmentation and high storage requirements, we propose a new marking scheme to further decrease the storage requirements for a router. As shown in Table 1, we use the 16-bit ID field as our marking field in our traceback scheme. While we keep low storage requirements, our storage can still be bounded by path numbers and the fragmented packets can be reassembled.

**Figure 1:**
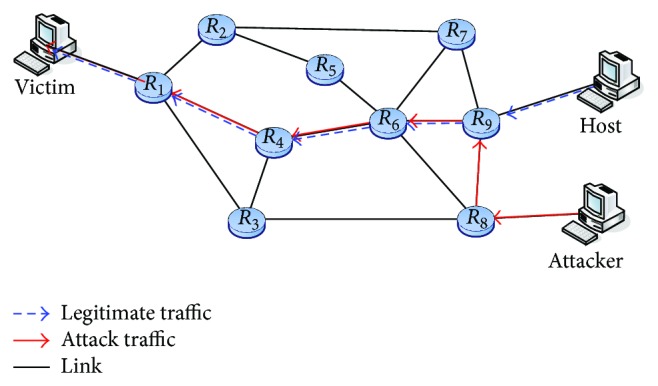
An example setup of our traceback scheme.

Figure 1 illustrates an example setup of our traceback scheme. A router can be connected to a local network or other routers. Here we assume there are *y* routers in the set *R*, that is, *R* = {*R*_1_, *R*_2_,…, *R*_*i*_,…, *R*_*y*_}, and each router complies with our protocol. A border router receives packets from its local network and sends the packets to the destination through a core router. Because packets come from different sources, a border router may also be a core router. For example, *R*_9_ serves as a border router when it receives packets from Host. However, it becomes a core router when receiving packets from *R*_8_. In the following discussion, we use *D*(*R*_*i*_) to indicate the degree of router *R*_*i*_, that is, the number of routers adjacent to *R*_*i*_. But the degree does not include the interface of a LAN. Besides, we require each router to maintain an interface table, in which UI_*i*_ denotes the upstream interface number of router *R*_*i*_ and 0 ≤ UI_*i*_ ≤ *D*(*R*_*i*_) − 1.

In our protocol, any router *R*_*i*_ and its network topology has to follow the following assumptions:

- *R*_*i*_ is secure against attacks,
- *R*_*i*_ maintains an interface table, in which the interface number ranges from 0 to *D*(*R*_*i*_) − 1,
- routers on an attack route, from the attack source to the victim, have stable interface tables and degrees during path reconstruction,
- *R*_*i*_ knows whether a packet comes from a router or from a local network,
- this traceback scheme is viable on every router. If there are any routers unable to comply with this scheme, they can establish a tunnel to communicate with each other.

The notations used in our protocol are listed in Notations section.

Our traceback scheme consists of two stages: the first is marking/logging stage, and the second is path reconstruction. The steps of how we trace the origin of an attack will be elaborated in the following subsections.

### 2.1. Marking and Logging

In our marking scheme, we mark a router' interface numbers and store the mark in a packet's IP header. But an IP header has only limited space, so we combine logging with marking to log marks on the routers. During path reconstruction, each router can only track its upstream router's adjacent interface number.

When a packet enters a network from its host, every router that complies with our protocol has to mark its own route info on the passing packets and store the mark in each packet's marking field. Each router's route info consists of the interface number where the packet enters; its log table's information; and its degrees. The packets that a router receives can be categorized into two types. In the first type, when a border router receives a packet from its local network, it sets the packet's marking field as zero and forwards the packet to the next core router. Therefore, when adversaries send attack packets with a forged path in the marking field trying to confuse our tracking, we can still locate their origin correctly. Hence we can verify whether a router is the source router of an attack by checking if the marking field is zero. On the other hand, when a core router *R*_*i*_ receives a packet *P*_*j*_ from another router, *R*_*i*_ uses packet *P*_*j*_'s mark *P*_*j*_ · mark and the incoming interface UI_*i*_ and the degree *D*(*R*_*i*_) to compute a new marking field mark_new_ = *P*_*j*_ · mark × (*D*(*R*_*i*_) + 1) + UI_*i*_ + 1. If mark_new_ does not overflow, that is, ≤65535, the core router *R*_*i*_ overwrites *P*_*j*_ · mark with mark_new_ and then forwards the packet to the next router. If mark_new_ overflows, that is, >65535, the core router *R*_*i*_ has to log the mark into a log table and use the index value to calculate a new mark_new_. However, such a marking and logging method may require more log tables on a router. According to CAIDA's skitter data [29], this method would exceed a log table's maximum entries [26].

As shown in Table 2(a), a router's log table *HT*_*k*_ consists of three parts: the top row is used to indicate the table's creation time *T*_*k*_^*s*^ and fill-up time *T*_*k*_^*f*^; the left column indicates the index of each entry; the right column stores packets' marks. The marks include the routers' interface numbers and are passed to the next router with the packets. But a large degree *D*(*R*_*i*_) makes a large logged mark, which can cause high logging frequency and increase the storage requirements for its downstream routers. To lower the logging frequency caused by a large interface number UI_*i*_, router *R*_*i*_ logs the packet mark and its interface number UI_*i*_ to reduce the mark size; see Table 2(b). However, if we insert the interface number into a logging table, it requires more storage for router *R*_*i*_ to store the table. To balance the storage requirements for router *R*_*i*_ and its downstream routers and to have lower average global storage requirements, we set a threshold for a router's degree so as to decide whether to write the interface number UI_*i*_ into a packet's header or into a log table. We suggest the value of threshold in Section 3.

Algorithm 1 shows the detailed steps of our marking and logging scheme. When a router receives a packet *P*_*j*_ and needs to log its mark, the router checks its degree *D*(*R*_*i*_) to decide whether or not to log the interface number UI_*i*_; compare lines 29–33 in Algorithm 1. If its *D*(*R*_*i*_) < threshold, the router chooses the log table *HT*_*k*_ by hashing the packet's *P*_*j*_ · srcIP to calculate the table number *k* = *H*_table_(*P*_*j*_ · srcIP); compare line 6 in Algorithm 1. Next, the router checks if there is any logged mark in *HT*_*k*_ identical with *P*_*j*_ · mark, searching from entry index *l* = 1 in monotonic ascending order to the largest index value; compare lines 17–19 in Algorithm 1. If the router cannot find a matched mark in the table, it logs *P*_*j*_ · mark into the empty entry that has the smallest index value; compare line 26 in Algorithm 1. Because we have set a threshold value, when *D*(*R*_*i*_) < threshold, our protocol has to write the interface number UI_*i*_ into the packet's mark instead of having it logged. This part is the main difference between our marking/logging and HAHIT's [26], and it is aimed at preventing data collision and waste of table size in HAHIT.

Then, the router uses the entry's index *l* and the packet's incoming interface UI_*i*_ to compute a new mark mark_new_ = ((UI_*i*_ ≪ *l* · length) + *l*) × (*D*(*R*_*i*_) + 1) and sends the new mark to the downstream router; compare line 32 in Algorithm 1. When *D*(*R*_*i*_) < threshold, the router searches the table, from *l* = 1 to the largest index value in monotonic ascending order, to check if there is a logged mark and a logged UI that are identical with *P*_*j*_ · mark and UI_*i*_; compare lines 13–15 in Algorithm 1. If it cannot find a matched mark and UI, it logs *P*_*j*_ · mark and UI_*i*_ as a pair into the empty entry that has the smallest index value; compare lines 13–15 in Algorithm 1. Since UI_*i*_ is now logged, here we only use the index value *l* to compute a new mark mark_new_ = *l* × (*D*(*R*_*i*_) + 1); compare line 30 in Algorithm 1. Next the router sends the new mark to the downstream router.

Since which table will be used to log a packet is determined by the hash value of the packet's source, packets that have the same source IP but come from different routes will be logged in the same table [26]. A large table leads to large index values and large marks, which will cause high logging frequency in the downstream routers. But a logging table with limited size will be filled up quickly if we use a hashed source IP to determine the table number. To prevent the problem of insufficient table entries, we create a new table when the table is full. We use each table's top row [*T*_*k*_^*s*^, *T*_*k*_^*f*^) to indicate the table's creation time *T*_*k*_^*s*^ and fill-up time *T*_*k*_^*f*^, in which *T*_*k*_^*s*^ < *T*_*k*_^*f*^ and *T*_*k*_^0^ is the first table's creation time. Therefore, every log table's top row is initialized as [*T*_*k*_^0^, *T*_*k*_^*∞*^). The analysis of the threshold value, the log table's size and the log table's numbers, and how they affect the storage requirements for one single router or for all the routers will be provided in Section 3.

**Figure 2:**
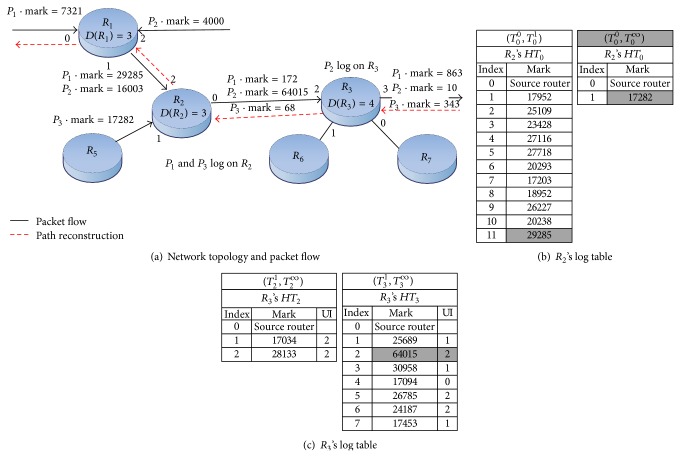
Example of our traceback.

Figure 2(a) exemplifies our marking and logging scheme. In the figure, packets *P*_1_, *P*_2_, and *P*_3_ come from different sources and their marks are logged on routers *R*_2_ and *R*_3_, whose threshold = 3. The grey cells in Figure 2 indicate the indexed entries of the log tables. When *P*_1_ passes through *R*_1_ en route for *R*_2_, its mark is larger than 65535. The router uses *P*_1_'s source IP to calculate *k* = *H*_table_(*P*_1_ · srcIP) = 0, so the mark will be logged into the table *HT*_0_. Then it goes to the cell whose index value *l* = 1, that is, *HT*_0_^1^ · mark, and compares the stored mark with *P*_1_ · mark. Because the router's degree is only 3, it searches the table from *l* = 1 to *l* = 11. As the router cannot find any mark that matches *P*_1_ · mark, it logs *P*_1_ · mark into *HT*_0_^11^.

After packet *P*_2_ passes through the routers *R*_1_ and *R*_2_, it enters *R*_3_ and needs to be logged. The router uses *P*_2_'s source IP to compute *k* = 3 and therefore the mark will be logged into the table *HT*_3_. However, as Figure 2(c) shows, after searching the table *R*_3_ finds that *P*_2_ · mark and *HT*_3_^2^ · mark are identical and the interface that *P*_2_ enters is the same as *HT*_3_^2^ · UI. It means this route has been taken by other packets and it has been logged in the table. Since the mark that matches *P*_2_ · mark is logged in the cell *HT*_3_^2^, we use *l* = 2 and *D*(*R*_3_) = 4 to compute a new mark mark_new_ = 2 × (4 + 1) = 10.

When *P*_3_ needs to be logged into *R*_2_'s *HT*_0_ but *HT*_0_ has reached its storage limit, the table's fill-up time will be changed to the present time *T*_0_^1^. A new log table will be created and its creation time will be set as *T*_0_^1^, denoted as _1_^*∞*^ *HT*_0_^1^. Last, *P*_3_ · mark will be logged into the new table's first entry _1_^*∞*^ *HT*_0_^1^.

### 2.2. Path Reconstruction

As shown in Algorithm 2, when a victim detects *P*_*j*_ as an attack packet at the time *T*_*r*_, it sends *P*_*j*_ and *T*_*r*_ to the tracking server and requests the server to find the attack source. The server sends the packet's mark *P*_*j*_ · mark, source IP *P*_*j*_ · srcIP, and the reception time *T*_*r*_ to the victim's upstream routers. When each router receives the request for path reconstruction, it uses *P*_*j*_ · mark to compute UI_*i*_; compare line 1 in Algorithm 2. If UI_*i*_ is not −1, it means the packet has never been logged on this router. Then the router uses the received mark to compute mark_old_ = *P*_*j*_ · mark/(*D*(*R*_*i*_) + 1) and sets *P*_*j*_ · mark as mark_old_; compare lines 34-35 in Algorithm 2. Next, it sends the request to its upstream router that is adjacent to UI_*i*_; compare line 35 in Algorithm 2. If a router's UI_*i*_ is −1, it means the packet has been logged on this router. If the router's degree does not exceed the threshold, the router divides *P*_*j*_ · mark by (*D*(*R*_*i*_) + 1) and obtains the index value *l* and UI_*i*_; compare lines 6-7 in Algorithm 2. If *l* ≠ 0, it uses *P*_*j*_ · srcIP to find which table *P*_*j*_ · mark is logged in. It computes *k* = *H*_table_(*P*_*j*_ · srcIP); compare line 10 in Algorithm 2. Because the current log table may not be the one that was used to log⁡⁡*P*_*j*_ · mark, the router has to use *T*_*r*_ to find the log table whose time field matches *T*_*r*_. After the table number and its time field are verified, the router follows the index value *l* to retrieve mark_old_ from table *k*; compare lines 16 and 26 (if table is full) in Algorithm 2. Because of our setup of a threshold value, when *D*(*R*_*i*_) < threshold, our path reconstruction has to take UI_*i*_ and *l* into consideration. And this part has made the major difference between our path reconstruction and HAHIT's [26]. If the router's degree *D*(*R*_*i*_) is larger than the threshold, it uses the mark *P*_*j*_ · mark to compute *l* = *P*_*j*_ · mark/(*D*(*R*_*i*_) + 1); compare line 4 in Algorithm 2. Likewise, if *l* ≠ 0, it computes the table number *k* (cf. line 10 in Algorithm 2) and checks the table's time field to find the table that logs the mark and the interface number. Last it follows *l* to retrieve mark_old_ and UI_*i*_ from table *k*; compare lines 13-14 and 23-24 (if table is full) in Algorithm 2. But if *l* = 0, it means this router is the source router; compare line 31 in Algorithm 2.

In Figure 2, we use dotted lines to indicate the path reconstruction of packet *P*_1_. It shows *R*_3_ receives the path reconstruction request in which *T*_0_ < *T*_*r*_ < *T*_1_. After *R*_2_ receives *P*_1_ · mark, *P*_1_ · srcIP, and *T*_*r*_ from *R*_3_, it uses *P*_1_ · mark to compute UI_2_ = −1, which means the packet has been logged on *R*_2_. Since the router's degree is three, the router divides *P*_1_ · mark by (*D*(*R*_2_) + 1) and retrieves the pair of data *l* = 11 and UI_2_ = 2. As *l* ≠ 0, this router is not the source router. Then it uses *P*_1_ · srcIP to compute the log table's number *k* = 0. According to *T*_0_^0^ < *T*_*r*_ < *T*_0_^1^, the router retrieves the mark from _0_^1^ *HT*_0_^11^ and replace *P*_1_ · mark with the retrieved mark. Last, *P*_1_ · mark is sent to *R*_1_ to continue the traceback until the attack source is identified.

## 3. Performance Analysis

In this section, we will introduce our simulation environment and how we determine log table size and the threshold.

### 3.1. Simulation Environment

**Figure 3:**
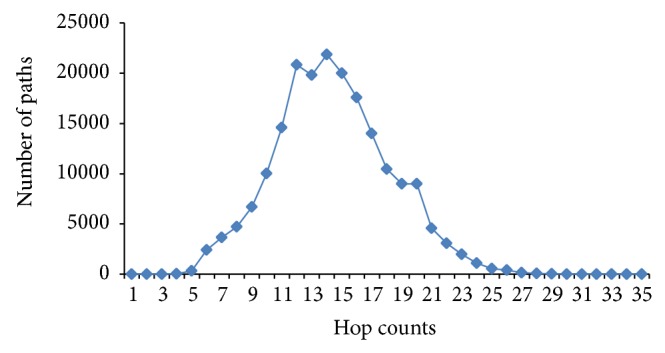
Distribution of path length.

To simulate the Internet topology, we use the skitter project topology distributed by CAIDA [29] as our sample data set of the Internet. The data set consists of paths to a specific host of the topology. We analyze CAIDA's skitter data and choose only 197,003 complete paths for our network topology. The analysis results are illustrated in Figure 3. Total number of its routers is 130,267; its average hop count of paths is 14.42; and its average upstream degree is 2.63.

### 3.2. Relation between Router Degree and Table Size

**Figure 4:**
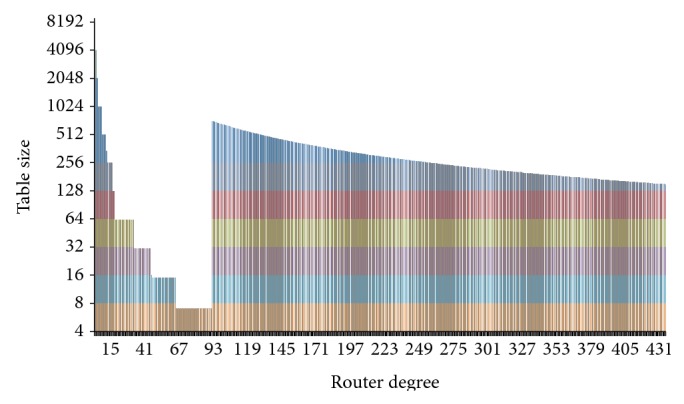
Relation between router degree and table size.

As shown in Figure 4, when a router's degrees are below 90, the table's maximum size decreases quickly with the increase of router degrees. It is because when the router's degrees are under the threshold our scheme marks the router's interface number UI_*i*_ into the fixed-size packet header. When UI_*i*_'s maximum number increases with the degree, the index value has to decrease. It means the maximum size of the table decreases too. When the degrees are over 90, UI_*i*_ has to be logged in the table and therefore the marking field allows a higher index value. This is why a log table's maximum size rises drastically when the router's degrees are larger than 90. For example, if a router's degrees are 66, the maximum size of its log tables is 7. The router can only accommodate log tables whose size ranges from 4 to 7. If the degrees are 91, the router allows log tables whose maximum size is 712. Then the table's maximum size decreases with the increase of degrees.

### 3.3. Relation among Threshold, Table Size, and Logging Times

**Figure 5:**
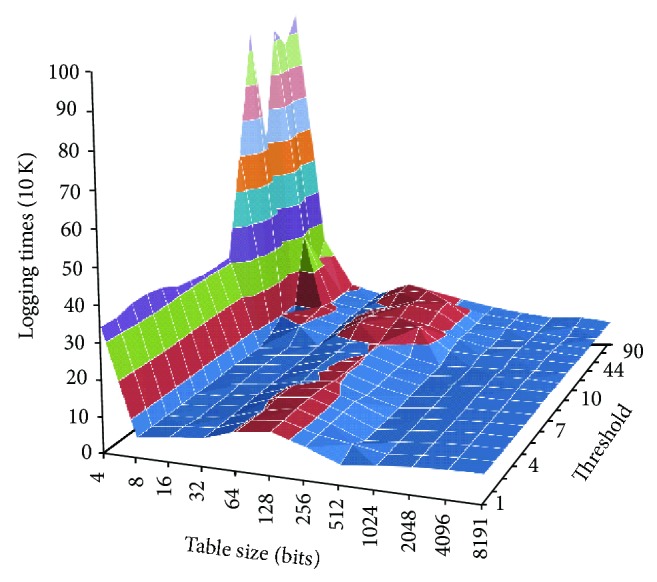
Relation among threshold, table size, and logging times.

Since the logging algorithm is determined by the threshold of a router's degree, we send 10 million packets to the network to find out the maximum storage requirement of our scheme. In the simulation, we send the packets to a randomly chosen path and count the logging times on the largest router in CAIDA's dataset, whose degree is 434. The result is shown in Figure 5. When the threshold is set as 10, the table has 8 entries (256 bits) and the router has the fewest logging times. Therefore, we suggest that routers set the table's maximum size as 256 bits and the threshold 10.

### 3.4. Storage Requirements

**Figure 6:**
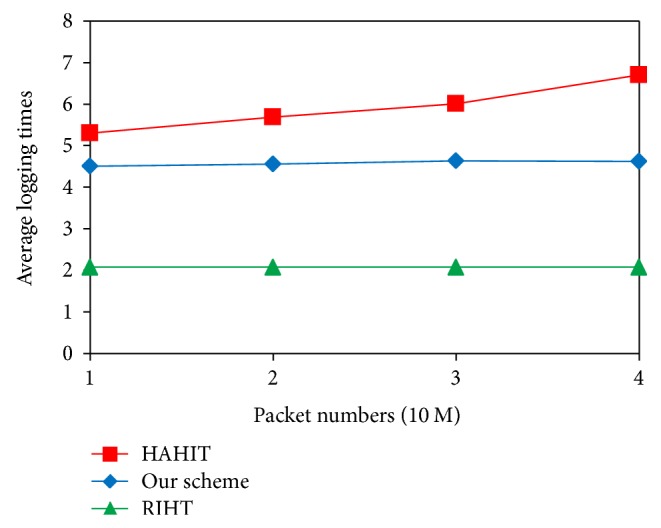
Comparison of logging times.

In this subsection, we compare the logging times and storage requirements of our scheme with those of other traceback schemes RIHT [24] and HAHIT [26].  Figure 6 shows the average logging times of our scheme and of RIHT and HAHIT when we send 10–40 million packets to the network. As depicted in the figure, compared with HAHIT our scheme requires fewer logging times and our logging times do not increase with the number of packets. Since the size of a marking field is fixed, a large index will leave a small space for the packet mark. And this can cause higher logging frequency. But our scheme requires an interface number to be logged if it exceeds the threshold value. In doing so, we can effectively lower the logging frequency. Furthermore, our logging frequency does not linearly increase with packet numbers because the index value of our scheme is bounded by the threshold. As for RIHT, it has lower logging frequency than our scheme because its marking field requires 32 bits and therefore has lower chance of overflow. But this advantage declines with the increase of hops between source and destination. Besides, RIHT has higher false positive/negative rates.

**Figure 7:**
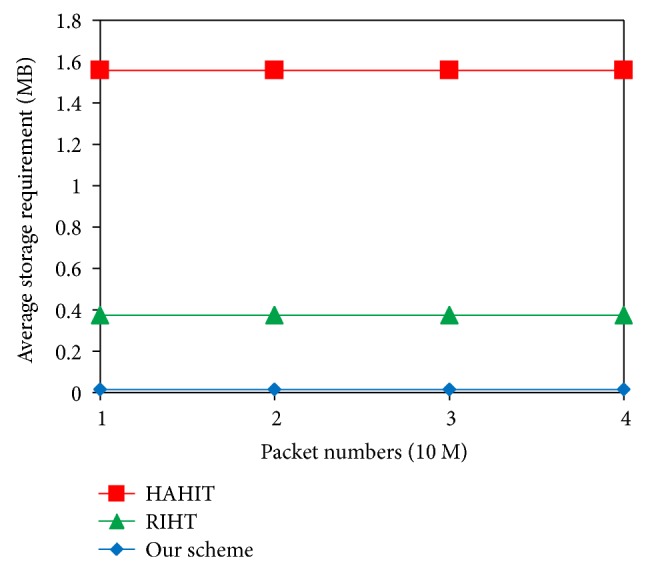
Comparison of average storage requirements.

Figure 7 shows the average storage requirements on each router. While HAHIT requires 1500 KB and RIHT 320 KB in average for their logging, ours only needs 16 KB. Although our logging frequency is slightly higher than RIHT's, our scheme cuts its storage requirement by 95%. It is because our log tables allow more entries on the routers whose degrees are under the threshold value 10, and because we do not use fixed-size tables. Thus, we can avoid the paths that have been logged twice in the tables.

**Figure 8:**
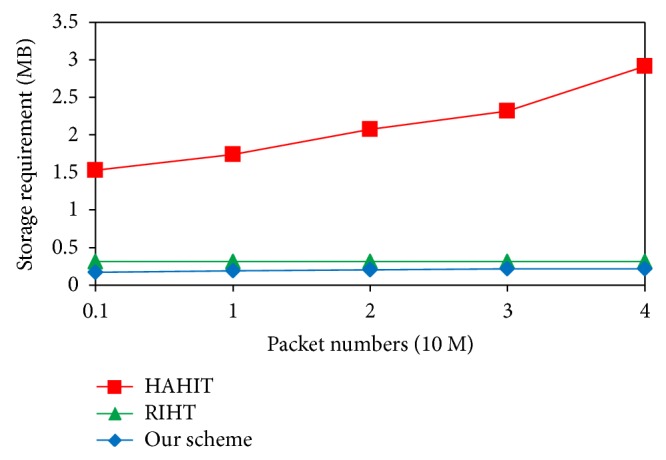
Comparison of the maximum storage requirements.

Figure 8 compares our scheme's maximum storage requirements with HAHIT's and RIHT's in different packet numbers. In these schemes, the maximum storage occurs on the router that has the largest degrees because it will have the highest logging frequency. Figure 8 shows our storage requirements and RIHT's storage requirements do not linearly increase with packet numbers because they have constant logging frequency. The maximum storage requirement of our scheme is 220 KB, about 2/3 of RIHT's, that is, 320 KB, because our scheme has smaller index values.

### 3.5. Computational Loads

**Figure 9:**
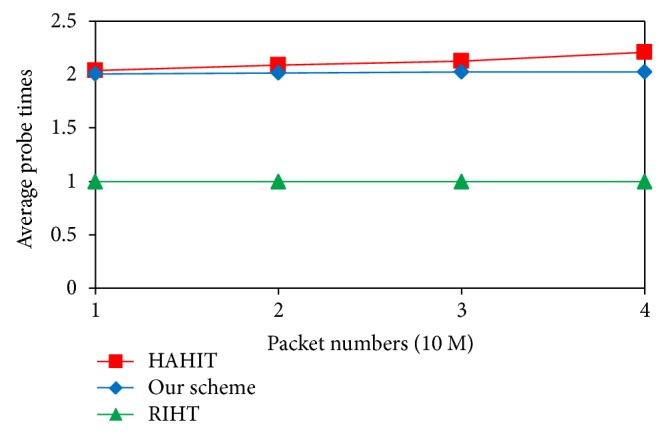
Average probe times in path reconstruction.

Since our scheme, HAHIT, and the RIHT use similar approaches to log packets, they have almost equal computation loads in logging. Thus, we analyze the computational loads of their path reconstruction only in this subsection. Besides, these three methods use packet marks to compute their log table's index value and then use the value to compute a new mark. Therefore, we analyze and compare the computation times required for each scheme to generate a valid index value.  Figure 9 shows RIHT needs only one computation to find a logged path because it has just one table. But HAHIT and our scheme have to find the log table first and then the index value, hence two probes at least. The probe numbers will slightly increase if we take into account the probes of those filled-up tables.  Figure 9 shows that compared with HAHIT, our average probe times are closer to 2. It is because the routers in our scheme with *D*(*R*_*i*_) > 10 use larger log tables to lower the chance of tables being filled up.

### 3.6. False Positive/Negative Rates

**Figure 10:**
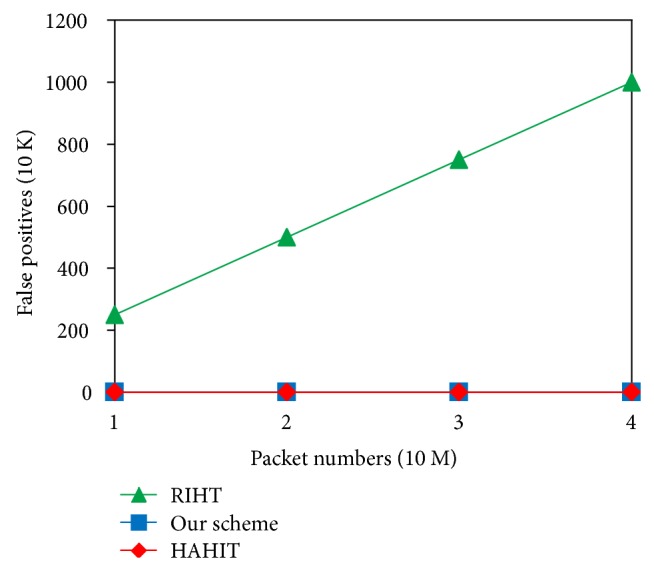
Comparison of false positives.

When a router is mistaken as an attack router, we call it a “false positive.” When we fail to trace back to an attacker, we call it a “false negative.” Besides, a router's storage capacity is limited. If packet numbers exceed a router's storage limit, its log tables have to be refreshed. Then false negatives may occur in path reconstruction. In our simulation, each time we only choose one host from CAIDA's dataset to act as an attacker sending one packet, and then we repeat the process 10–40 million times, so as to try the false positive/negative rates in RIHT, HAHIT, and our scheme. Because our scheme, HAHIT, and RIHT have low storage requirements, routers can keep the path info for a long time and therefore do not need to refresh their log tables under flood attacks, hence 0 false negatives. RIHT, however, requires 32 bits for marking and consequently cannot make 0 false positives. Its false positive rates equal its fragmentation rates 0.25% [26]. Since our scheme and HAHIT use 16-bit marking fields, our ID fields can remain intact in packet fragmentation. Thus, both of the two schemes can make 0 false positives. As shown in Figure 10, RIHT's false positives rise with the increase of packet numbers, while ours and HAHIT's still remain 0.

To sum up, we list the comparison results in Table 3.

## 4. Conclusion

In this paper we propose a 16-bit single packet IP traceback scheme. Compared with current hybrid single packet traceback schemes, it has the lowest maximum storage requirement, which means the compulsory storage requirement for a router to support our hybrid single packet traceback. Besides, our scheme stores user interface information on small-degree routers, so that it can have small average storage requirements. Because the required storage for our routers' log tables is bounded by route numbers, it does not grow with the number of passing packets. Our experiment also shows that our scheme cuts RIHT's average storage requirement (320 KB) by 95%. Despite one more probe required for our path reconstruction, if compared with RIHT's, our traceback can achieve high accuracy (zero false positive/negative rates) because it complies with IPsec preventing marked packets from being dropped by routers during logging. Last, our scheme guarantees reassembly of fragmented packets at the destination.

## Figures and Tables

**Algorithm 1:**
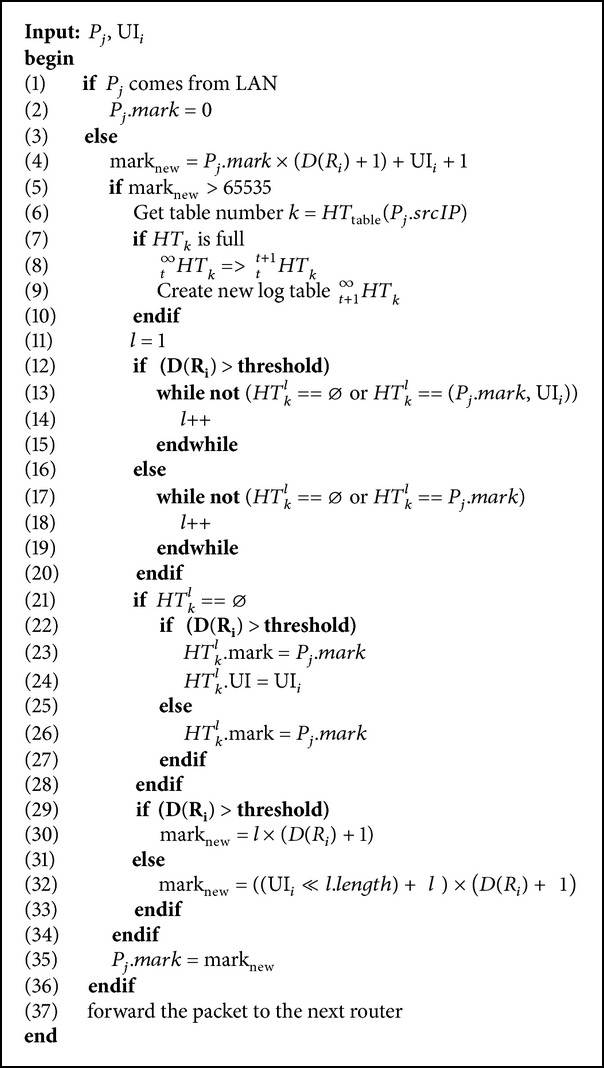
Our marking and logging scheme.

**Algorithm 2:**
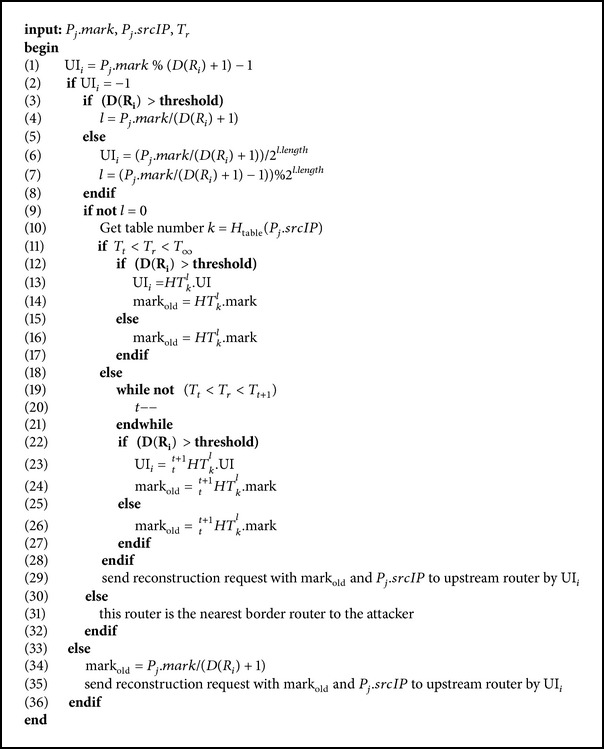
Our path reconstruction.

**Table 1:** Our marking field in an IP header (the bold text).

Bit offset	0–3	4–7	8–15	16–18	19–31

0	Version	Header length	TOS	Total length	

32	**Identification field**			Flag	Fragment offset

64	TTL		Protocol	Header checksum	

96	Source address				

128	Destination address				

160	Options				

160 Or 196+	Payload (first 8 bytes)				

**Table:** (a) If *D*(*R*_*i*_)≤ threshold

[*T*_*k*_^*s*^, *T*_*k*_^*f*^)	
*HT*_*k*_	
Index	Mark
0	Source router
⋮	⋮
*l*	*P*_*j*_ · *mark*
⋮	⋮

**Table:** (b) if *D*(*R*_*i*_) > threshold

[*T*_*k*_^*s*^, *T*_*k*_^*f*^)		
*HT*_*k*_		
Index	Mark	UI
0	Source router	
1	*P*_*g*_ · *mark*	*P*_*g*_ · UI_*i*_
⋮	⋮	⋮
*l*	*P*_*j*_ · *mark*	*P*_*j*_ · UI_*i*_

**Table 3:** Comparison results.

	Marking size	Storage requirement	Major contributions
RIHT	32 bits	320 KB	RIHT bounds the storage requirements but may be prone to a fragmented traffic.

HAHIT	16 bits	1500 KB and above	HAHIT prevents fragmentation issues by changing the marking field from 32 bits to 16 bits, but its storage requirement is higher and its table search is inefficient.

Our scheme	16 bits	220 KB	Our scheme sets a threshold to determine whether to log UI or to mark UI in a packet, so as to solve the storage and fragmentation issues at the same time.

## Notations

$R_{i}$: $\left\{R_{1},R_{2},\ldots ,R_{i},\ldots ,R_{x}\right\}$, routers in a network
$D(R_{i})$: The degree of $R_{i}$, that is, the number of $R_{i}$’s adjacent routers
$P_{j}$: A packet j
${\text{UI}}_{i}$: The upstream interface number of router $R_{i}$
$P_{j}\cdot \text{mark:}$: Marking field of $P_{j}$
$P_{j}\cdot \text{srcIP:}$: $P_{j}$’s source IP
m: A log table with m entries
$H_{\text{table}}(\cdot )$: A hash function with hashed value ranging from 0 to n-1, where n denotes the number of log tables
$\left[T_{k}^{s},T_{k}^{f}\right)$: $T_{k}^{s}$ denotes log table k’s created time; and $T_{k}^{f}$ denotes k’s full time, where s, f=0…,t,…,∞. If s=0, it means the first table of k. If f=∞, it means the table has not been filled up
HTtt+1kl: The log table k, in which l denotes the table’s index value ranging from 0 to m-1. If l=0, it refers to the source router. A packet’s marking field is logged at ${HT}_{k}^{l}\cdot \text{mark}$. ${\text{UI}}_{i}$ is logged at ${HT}_{k}^{l}\cdot \text{UI}$. HTtt+1k denotes table k’s creation time is $T_{t}$ and fill-up time is $T_{t+1}$. If the fill-up time is not specified, table k must be currently in use and is denoted as HTt∞k
%: Modulo operation.
